# Accuracy of Transcutaneous Carbon Dioxide Measurement in Premature Infants

**DOI:** 10.1155/2016/8041967

**Published:** 2016-06-08

**Authors:** Marie Janaillac, Sonia Labarinas, Riccardo E. Pfister, Oliver Karam

**Affiliations:** Neonatal and Pediatric Intensive Care Unit, Geneva University Hospital, Rue Willy Donzé 6, 1250 Geneva, Switzerland

## Abstract

*Background.* In premature infants, maintaining blood partial pressure of carbon dioxide (pCO_2_) value within a narrow range is important to avoid cerebral lesions. The aim of this study was to assess the accuracy of a noninvasive transcutaneous method (TcpCO_2_), compared to blood partial pressure of carbon dioxide (pCO_2_).* Methods.* Retrospective observational study in a tertiary neonatal intensive care unit. We analyzed the correlation between blood pCO_2_ and transcutaneous values and the accuracy between the trends of blood pCO_2_ and TcpCO_2_ in all consecutive premature infants born at <33 weeks' gestational age.* Results.* 248 infants were included (median gestational age: 29 + 5 weeks and median birth weight: 1250 g), providing 1365 pairs of TcpCO_2_ and blood pCO_2_ values. Pearson's *R* correlation between these values was 0.58. The mean bias was −0.93 kPa with a 95% confidence limit of agreement of −4.05 to +2.16 kPa. Correlation between the trends of TcpCO_2_ and blood pCO_2_ values was good in only 39.6%.* Conclusions.* In premature infants, TcpCO_2_ was poorly correlated to blood pCO_2_, with a wide limit of agreement. Furthermore, concordance between trends was equally low. We warn about clinical decision-making on TcpCO_2_ alone when used as continuous monitoring.

## 1. Background

The partial pressure of carbon dioxide (pCO_2_) strongly influences cerebral perfusion in premature infants that have reduced autoregulation capacity. Thus, monitoring pCO_2_ to avoid hyper- or hypocapnia and associated brain injury has become standard of care [1, 2]. Although some studies advocate “permissive hypercapnia” to limit lung injury due to mechanic ventilation, this attitude remains controversial [3]. In any case, most neonatologists consider it best practice to closely monitor pCO_2_ in premature infants, especially when they are ventilated.

Currently, the gold standard to measure tissue pCO_2_ is the blood gas analysis. However, this method has disadvantages, as it might contribute to spoliative anemia and is painful if done by capillary sampling. Therefore, other pCO_2_ monitoring methods have been developed during the last three decades [4].

End-tidal CO_2_ measurement provides a noninvasive estimate of the blood pCO_2_ in ventilated patients. However, its use in premature infants is limited by the small tidal volumes and increased dead space caused by the measurement device inserted into the ventilation circuit. Furthermore, it is impossible or unreliable to use during alternate ventilation methods such as high frequency oscillation ventilation [5] or spontaneous breathing with or without continuous positive airway pressure.

Transcutaneous pCO_2_ (TcpCO_2_) was developed in the 1960s. A calibrated skin sensor, heated to 40–45°C (100–110°F) in order to arterialize the capillary bed and facilitate CO_2_ diffusion, can then measure the pCO_2_ in a thin liquid film between skin and sensor. Such devices are widely used in neonatal intensive care settings [6]. Several small studies in neonatal populations (the largest including 60 patients) have analyzed the correlation and concordance between the TcpCO_2_ and blood pCO_2_ [7–11]. The quality of the concordance between the two methods remains debated. One study in healthy adults has shown that very short-term trends appear accurate, with a mean TcpCO_2_ lag of about one minute after acute changes in arterial pCO_2_ [12]. However, no published study investigated the accuracy of TcpCO_2_ trends over longer periods.

The objective of our study was to assess, in a large neonatal population, the accuracy point values as well trends of TcpCO_2_ compared to blood pCO_2_.

## 2. Methods

This retrospective observational study included all consecutive premature infants born <33 weeks of postmenstrual age, admitted to our tertiary Neonatal Intensive Care Unit (NICU) at Geneva University Hospital over a period of four years starting from 1/1/2009.

All data during the first 28 days of life of these neonates were prospectively recorded in our electronic clinical data system (CliniSoft®, General Electric Healthcare, Milwaukee, WI, USA).

### 2.1. Blood pCO_2_ Determinations

The blood pCO_2_ was immediately analyzed after sampling on a Radiometer ABL800 analyzer (Radiometer, Brønshøj, Denmark). All results were automatically retrieved into our clinical data system including the time of determination. As several studies suggest that arterial, venous, and capillary blood pCO_2_ levels can be considered identical [13, 14], we did not differentiate the type of blood sample.

### 2.2. Transcutaneous pCO_2_ (TcpCO_2_) Determinations

All TcpCO_2_ sensors (TCM4®, Radiometer, Copenhagen, Denmark) were applied according to the manufacturer's recommendations and to our unit protocol: the sensor was calibrated every two to four hours, the skin cleaned with sterile water, two to three drops of specific contact gel were applied, and sensor was placed on the inside of the infant's thigh (on alternate sides, after each recalibration). The sensor temperature was set according to the manufacturer's guidelines (41°C for infants 500 g to 750 g and 42°C for infants 751 g to 2000 g). The membrane was changed every 14 days.

At least once an hour, the nurse in charge of the patient manually entered the TcpCO_2_ value into the electronic clinical data system.

### 2.3. Data Analysis

We considered pairs of TcpCO_2_ and blood pCO_2_ when both values were measured within an interval of 10 minutes.

The accuracy of point values was then assessed by three methods:

(1) The proportion of values where the difference between TcpCO_2_ and blood pCO_2_ was less than 10% and its 95% confidence interval of agreement.
(2) The correlation between TcpCO_2_ and blood pCO_2_ values; we considered, a priori, a good correlation defined by a Pearson *R* > 0.8.
(3) A Bland-Altman plot, to report the mean bias and 95% confidence limits of agreement between the two methods [15]; TcpCO_2_ and blood pCO_2_ values were averaged across the range and the mean bias was calculated as the mean difference between TcpCO_2_ and blood pCO_2_, and the 95% confidence limits of agreement between the two methods were defined as 1.96 times the standard deviation of the mean difference between TcpCO_2_ and blood pCO_2_.

The accuracy of trends was assessed for all consecutive pairs of TcpCO_2_ and blood pCO_2_, when pairs were measured within a six-hour interval. The trends of both values were expressed in percentage of change. Concordance between TcpCO_2_ and blood pCO_2_ trends was defined, a priori, as good if the difference between the values was ≤10%, moderate if the difference was 11 to 20%, and poor if the difference was >20% (see examples in Table 1).

All statistical analyses were performed with SPSS version 20 for Mac (SPSS, Chicago, IL, USA).

### 2.4. Sample Size

We anticipated 75% of good concordance between trends of TcpCO_2_ and blood pCO_2_. To obtain a 95% confidence interval smaller than ±5% around the proportion of good concordance, we calculated that at least 288 trends for each method would be required. We planned to include as many patients as possible, over a four-year period (to avoid historical biases), as long as the required number of inclusions would be matched.

The ethics committee of Geneva University Hospital approved the study.

## 3. Results

From January 1st 2009 to December 31st 2012, 248 patients were consequently included in this study. The median gestational age was 29.5 weeks (IQR 27.6; 31.5) and the median birth weight was 1250 g (IQR 972; 1592). One-third of the infants were intubated at least once. There were 61 (24.6%) cases of proven sepsis, 8 (3.2%) of necrotizing enterocolitis grade 3, 8 (3.2%) of grade 3 or 4 intracerebral hemorrhage, and 8 (3.2%) of periventricular leukomalacia. Over this period, 44 patients died (17.7%). Among the survivors, 5 infants (2.1%) had severe bronchopulmonary dysplasia.

1365 pairs of TcpCO_2_ and blood pCO_2_ point values were analyzed. The median interval between TcpCO_2_ and blood pCO_2_ measures was 4 minutes (IQR 2; 7). The median number of tests per patient was 4 (IQR 2; 9).

**Figure 1:**
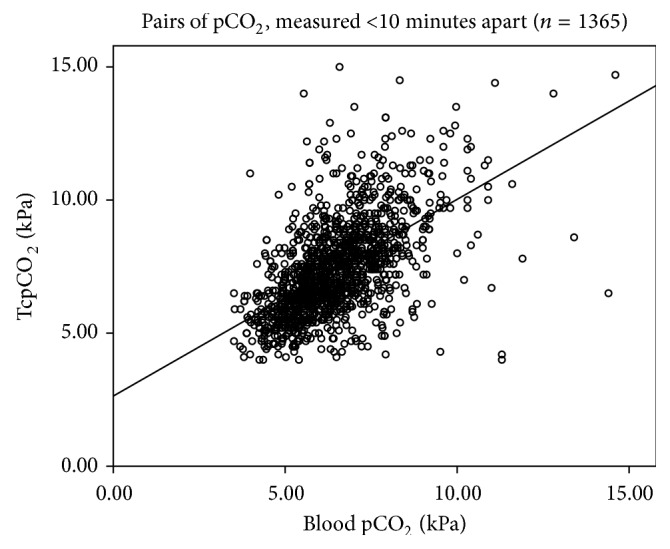
Scatter plot of the blood pCO_2_ and the TcpCO_2_ of values measured <10 mn. Scatter plot of the blood pCO_2_ (*x*-axis) and the TcpCO_2_ (*y*-axis) of values measured <10 minutes apart. Values are in kPa. *n* = 1365. Pearson's *R* = 0.58 (*p* < .001).

**Figure 2:**
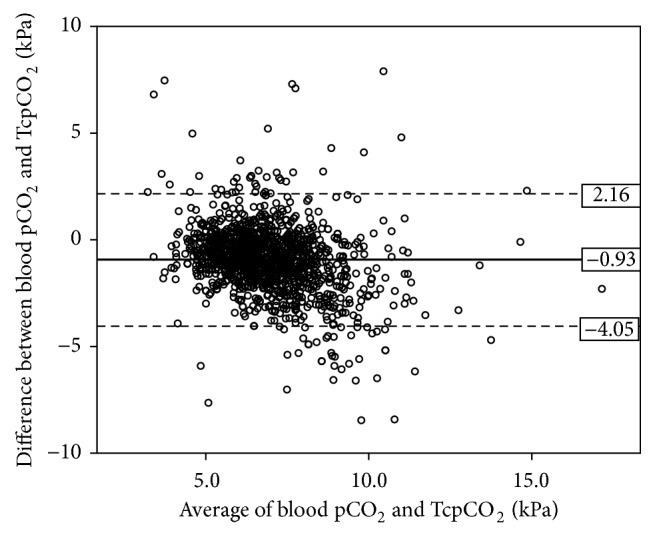
Bland-Altman plot of the average TcpCO_2_ and blood pCO_2_ values. Bland-Altman plot of the average TcpCO_2_ and blood pCO_2_ values (*x*-axis) and difference between TcpCO_2_ and blood pCO_2_ values (*y*-axis). *n* = 1365. Mean bias = −0.93 kPa and 95% confidence limit of agreement = −4.05 to +2.16 kPa.

The proportion of good concordance (less than 10% difference between TcpCO_2_ and blood pCO_2_) was 32.4% (442/1365, 95%CI 30.0; 34.9). Pearson's correlation coefficient between TcpCO_2_ and blood pCO_2_ was *R* = 0.58 (*p* < .001, Figure 1). The Bland-Altman analysis showed a mean bias of −0.93 kPa with a 95% confidence limit of agreement from −4.05 to +2.16 kPa (Figure 2). There was no specific pCO_2_ range with better precision.

**Figure 3:**
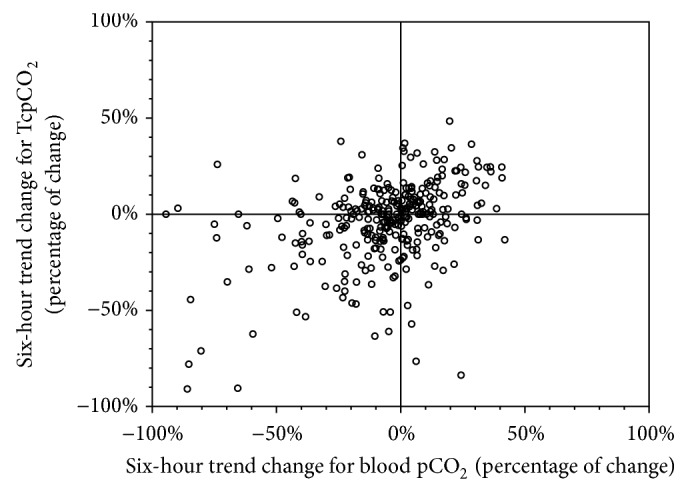
Scatter plot of the changes in blood pCO_2_ and the TcpCO_2_ six-hour trends. Scatter plot of the changes in blood pCO_2_ (*x*-axis) and the TcpCO_2_ (*y*-axis) six-hour trends. *n* = 313 pairs. Pearson's *R* = 0.25; *p* < .001.

There were 313 pairs of six-hour trends. The median number of pairs of trends per patient was 2 (IQR 1; 3). Pearson's correlation coefficient was *R* = 0.24 (*p* < .001, Figure 3). Good correlation between the trends (less than 10% difference) was observed in 39.6% (124/313, 95%CI 34.4; 45.1), moderate correlation (less than 20% difference) in 25.6% (80/313, 95%CI 21.0; 30.7), and bad correlation (more than 20% difference) in 34.8% (109/313, 95%CI 29.8; 40.3).

## 4. Discussion

Our results suggest that transcutaneous pCO_2_ with the TCM4® is not accurate in a general neonatal intensive care setting. The correlation between TcpCO_2_ and blood pCO_2_ was poor (Pearson's *R* = 0.58) and the 95% confidence limits of agreement wide (−4.05 kPa to 2.16 kPa) in the Bland-Altman analysis. The concordance between the six-hour trends of blood pCO_2_ and TcpCO_2_ was also poor, with only 39.6% of good concordance.

Several studies have analyzed the accuracy and the reliability of TcpCO_2_ monitoring, with conflicting results. Whereas some have described poor correlation between TcpCO_2_ and blood pCO_2_ [10, 16], others have shown better results [11, 17–19]. Kesten et al. have suggested very short-term trends, of a few minutes, to be accurate in healthy adults [12]. Two studies have described poor correlation blood pCO_2_ values for high values [7, 10].

Our study reports 1365 pairs of TcpCO_2_ and blood pCO_2_ in 248 different neonatal subjects, to assess the reliability of the noninvasive technique. Our Bland-Altman analysis is in line with a worsening correlation for high values (>10 kPa), but the rather low number of data points does not allow concluding in this matter (Figure 2). However, our study, with the largest number of matching samples published so far, strongly supports the studies with poor agreement between TcpCO_2_ and blood pCO_2_.

Our study is the first to report concordance between trends of TcpCO_2_ and blood pCO_2_ in premature infants. Indeed, although the point value correlation might be poor, most clinicians would rely on the trend of the TcpCO_2_ as a surrogate of the blood pCO_2_'s changes over time. Only one-third of the samples showed a good concordance of trends over a six-hour period.

It is legitimate to wonder if such monitoring may not lead to wrong clinical decisions or increased blood sampling to confirm values. Furthermore, as the sensor heats the skin to 41-42°C, it might cause discomfort and even injure the youngest infants' very fragile skin. Finally, the use of TcpCO_2_ is expensive. According to the manufacturer, the sensor needs recalibration every three to four hours using a specific gas cylinder. The skin fixation rings have to be relocated every 12 to 24 hours, and the sensor's membrane changed every one to two weeks. In our unit, the average yearly cost for one device used 24/7 is 10500$.

Some limitations to our study must be recognized. First, it is a retrospective study, and although the TcpCO_2_ values were recorded prospectively into the electronic clinical data system, it is impossible to retrospectively assess the skin's perfusion at the precise time. Second, our data system does not report the heating power or the sensor temperature or the precise site of use of the sensor. Therefore, we cannot ascertain the quality of each measured value. However, our nurses are instructed to use the probe according to the manufacturer's guidelines and to record the TcpCO_2_ value only when it has stabilized, and the large number of data points analyzed increases the credibility of our findings. Finally, the Bland-Altman analysis was not modified to adjust for multiple measures in the same patient [20]. However, the median of median number of repeated measures was low (4) compared to the number of patients (248). Furthermore, adjusting for multiple measures would only have increased the agreement limit. Despite the limitations, we would argue that our results represent very closely real life use of TcpCO_2_ and certainly those used in our own clinical practice to take decisions.

## 5. Conclusion

Our data show that TcpCO_2_ with the TCM4® poorly correlate to blood pCO_2_, with a wide confidence limit of agreement. A low concordance was also noted between trends of TcpCO_2_ and blood pCO_2_. We therefore warn about the sole use of TcpCO_2_ for clinical decision-making. Prospective, short-term correlations for value points and trends need to be assessed in further research as well as in nonneonatal populations.

## Figures and Tables

**Table 1:** Examples of concordance classification between blood pCO_2_ and transcutaneous pCO_2_.

Example	Blood pCO_2_			TcpCO_2_			Trends' difference	Trends' concordance
	*T*_0_	*T*_1_	Trend	*T*_0_	*T*_1_	Trend		
1	5.2	5.6	+7.7%	5.2	5.2	+0%	7.7%	Good
2	5.2	5.6	+7.7%	5.2	6.4	+23.1%	15.4%	Moderate
3	5.2	5.6	+7.7%	5.8	4.6	−21.4%	29.1%	Poor

Four examples of blood pCO_2_ and TcpCO_2_ trends within six-hour periods, with difference between trends, and the qualitative assessment of the concordance.

All pCO_2_ values are in kPa.

*T*_0_: measures at baseline.

*T*_1_: measures within a six-hour interval.

## Abbreviations

VLBW: Very low birth weight
pCO_2_: Partial pressure of carbon dioxide (pCO_2_)
TcpCO_2_: Transcutaneous partial pressure of carbon dioxide
NICU: neonatal intensive care unit.

## References

[1] Levene M. (2007). Minimising neonatal brain injury: how research in the past five years has changed my clinical practice. *Archives of Disease in Childhood*.

[2] Fabres J., Carlo W. A., Phillips V., Howard G., Ambalavanan N. (2007). Both extremes of arterial carbon dioxide pressure and the magnitude of fluctuations in arterial carbon dioxide pressure are associated with severe intraventricular hemorrhage in preterm infants. *Pediatrics*.

[3] Woodgate P. G., Davies M. W. (2001). Permissive hypercapnia for the prevention of morbidity and mortality in mechanically ventilated newborn infants. *Cochrane Database of Systematic Reviews*.

[4] Widness J. A., Madan A., Grindeanu L. A., Zimmerman M. B., Wong D. K., Stevenson D. K. (2005). Reduction in red blood cell transfusions among preterm infants: results of a randomized trial with an in-line blood gas and chemistry monitor. *Pediatrics*.

[5] Tobias J. D. (2009). Transcutaneous carbon dioxide monitoring in infants and children. *Paediatric Anaesthesia*.

[6] Molloy E. J., Deakins K. (2006). Are carbon dioxide detectors useful in neonates?. *Archives of Disease in Childhood: Fetal and Neonatal Edition*.

[7] Berkenbosch J. W., Tobias J. D. (2002). Transcutaneous carbon dioxide monitoring during high-frequency oscillatory ventilation in infants and children. *Critical Care Medicine*.

[8] Tingay D. G., Stewart M. J., Morley C. J. (2005). Monitoring of end tidal carbon dioxide and transcutaneous carbon dioxide during neonatal transport. *Archives of Disease in Childhood: Fetal and Neonatal Edition*.

[9] Martin R. J., Beoglos A., Miller M. J., DiFiore J. M., Robertson S. S., Carlo W. A. (1988). Increasing arterial carbon dioxide tension: influence on transcutaneous carbon dioxide tension measurements. *Pediatrics*.

[10] Hejlesen O. K., Cichosz S. L., Vangsgaard S., Andresen M. F., Madsen L. P. (2009). Clinical implications of a quality assessment of transcutaneous CO_2_ monitoring in preterm infants in neonatal intensive care. *Studies in Health Technology and Informatics*.

[11] Lopez E., Grabar S., Barbier A., Krauss B., Jarreau P.-H., Moriette G. (2009). Detection of carbon dioxide thresholds using low-flow sidestream capnography in ventilated preterm infants. *Intensive Care Medicine*.

[12] Kesten S., Chapman K. R., Rebuck A. S. (1991). Response characteristics of a dual transcutaneous oxygen/carbon dioxide monitoring system. *Chest*.

[13] Zavorsky G. S., Cao J., Mayo N. E., Gabbay R., Murias J. M. (2007). Arterial versus capillary blood gases: a meta-analysis. *Respiratory Physiology and Neurobiology*.

[14] Yildizdaş D., Yapıcıoğlu H., Yilmaz H. L., Sertdemir Y. (2004). Correlation of simultaneously obtained capillary, venous, and arterial blood gases of patients in a paediatric intensive care unit. *Archives of Disease in Childhood*.

[15] Bland J. M., Altman D. G. (1995). Comparing methods of measurement: why plotting difference against standard method is misleading. *The Lancet*.

[16] Aliwalas L. L. D., Noble L., Nesbitt K., Fallah S., Shah V., Shah P. S. (2005). Agreement of carbon dioxide levels measured by arterial, transcutaneous and end tidal methods in preterm infants ≤ 28 weeks gestation. *Journal of Perinatology*.

[17] Hand I. L., Shepard E. K., Krauss A. N., Auld P. A. M. (1989). Discrepancies between transcutaneous and end-tidal carbon dioxide monitoring in the critically ill neonate with respiratory distress syndrome. *Critical Care Medicine*.

[18] Geven W. B., Nagler E., de Boo T., Lemmens W. (1987). Combined transcutaneous oxygen, carbon dioxide tensions and end-expired CO_2_ levels in severely ill newborns. *Advances in Experimental Medicine and Biology*.

[19] Sørensen L. C., Brage-Andersen L., Greisen G. (2011). Effects of the transcutaneous electrode temperature on the accuracy of transcutaneous carbon dioxide tension. *Scandinavian Journal of Clinical and Laboratory Investigation*.

[20] Bland J. M., Altman D. G. (2007). Agreement between methods of measurement with multiple observations per individual. *Journal of Biopharmaceutical Statistics*.

